# Work-related risk factors and the prevalence of low back pain among low wage workers: results from a cross-sectional study

**DOI:** 10.1186/s12889-019-7430-9

**Published:** 2019-08-08

**Authors:** Sintayehu Daba Wami, Giziew Abere, Awrajaw Dessie, Dawit Getachew

**Affiliations:** Department of Environmental and Occupational Health and Safety, Institute of Public Health, University of Gondar, 196 Gondar, Ethiopia

**Keywords:** Low back pain, Hotel, Housekeeper, Prevalence, Risk factor, Ethiopia

## Abstract

**Background:**

Low back pain, the most commonly reported musculoskeletal problem, is a major burden on individuals, health systems and social care systems with the indirect cost being predominant. It results in disability, poor service, low quality of life and sickness absences in working places. The problem of low back pain and its risk factors among hotel housekeepers are not well known in Ethiopia. Therefore, this study was aimed to investigate the prevalence and identify determinants of low back pain among hotel industries’ housekeepers in Gondar town, Ethiopia.

**Methods:**

Institutional based cross-sectional study was conducted from March to May 2017. A systematic random sampling technique was applied to select 422 study participants, and the data was collected by a standardized Nordic questionnaire for the analysis of musculoskeletal symptoms. Bivariate and multivariable binary logistic regression analyses were performed using SPSS version 20. The significance level was obtained at 95% CI and *p*-value ≤ 0.05.

**Results:**

The prevalence of low back pain among hotel housekeepers in Gondar town was 58.1% (95% CI: 53.6, 62.8%). Being temporary employee (AOR: 3.22), type of job which requires reaching/overstretching (AOR: 2.93), engaging in a job that requires repetitive bending (AOR: 1.97), making > 30 beds per day (AOR: 3.19) signified the significant risk factors for low back pain. However, hotel housekeepers who were satisfied in their current job were less impacted by low back pain (AOR: 0.49).

**Conclusion:**

A high proportion of hotel housekeepers in this study reported they had low back pain. Employment pattern, rest break taken, reaching/overstretching, repetitive bending, job satisfaction, training related to health and safety and numbers of beds making were among the factors associated with low back pain. Hence, ergonomic measures focusing on correcting the arrangement of work station, rest breaks and changing some equipment are potentially important targets to reduce the problem.

## Background

Low back pain, the most commonly reported musculoskeletal problem, is a major burden on individuals, health systems and social care systems with the indirect cost being predominant [1, 2]. It has become a major public health problem among the working population in recent years. This problem results in disability, poor services and sickness absences in working places [3–5]. Musculoskeletal disorders (MSDs) are impairments of body structures such as muscles, joints, tendons, ligaments, nerves, bones and the localized blood circulation system. Moreover, back pain is defined as chronic or acute pain, aches or trouble in the lumbar or buttock area sometimes called lumbago, or in the upper leg region which is a major work-related disorder in almost all physically demanding jobs [6].

Housekeeping is a physically demanding job. It involves forceful movements and working in awkward body positions while lifting mattresses, cleaning tiles and vacuuming in every shift; which in turn contribute to MSDs [7, 8]. Hotel housekeepers are required to carry or move tons of trash and used linen every day. This makes it easy to see that many injury types are directly related to the tasks performed in housekeeping occupation [8, 9]. Housekeepers had the highest overall injury rate and the highest rate of MSDs, most commonly low back pain, among hotel industry workers in the United States [10, 11].

The 2010 Global Burden of Disease (GBD) study estimated that low back pain (LBP) is among the top 10 diseases and injuries that account for disability-adjusted life years worldwide [12]. The prevalence of LBP among hotel housekeepers has been shown to be high. The prevalence was believed to vary from 46 to 77% in different parts of the world [6, 9, 13–15].

Work-related LBP is associated with exposure to ergonomic stressors at work, environmental (physical), psychosocial and/or personal risk factors [16]. In many studies, a wide-range of factors associated with low back pain has been identified. Among these; lifting and carrying heavy objects [17], awkward posture, psychosocial job demands and job dissatisfaction [18], repetitive movement, static workload [6], bed making [9] were reported as a major risk factor for LBP. Other factors such as sleep problem [19], not doing regular physical exercise, dissatisfaction with working environment and culture [20], duration of employment, pulling and pushing heavy loads, bending and working with twisted trunks [21], alcohol consumption and lack of rest [22] were also noted to be a predictors of LBP.

Though there are several studies done on the magnitude and associated factors of back pain among different groups of the workforce, no study has been carried out among hotel housekeepers in Ethiopia. Therefore, this study was aimed to investigate the prevalence and identify determinants of low back pain among hotel industries’ housekeepers in Gondar town, Ethiopia.

## Methods

### Study design and setting

The institution-based cross-sectional study design was employed from March 01–May 20, 2017. The study was conducted in Gondar town hotels, Northwest Ethiopia. Gondar town is one of tourist destination city in Amhara Regional State, which is located in the northwest part of Ethiopia, about 750 Km from the capital city, Addis Ababa. The hotel industry is one of the known industries in the town and housekeepers are the largest workforce in this industry.

### Source and study population

All housekeepers who were employed in Gondar town hotels were the source population. About 1220 housekeepers were currently employed in Gondar city hotels. Housekeepers in Gondar town hotels, who had worked at least for 12 months in the hotels, were included in this study. While, hotel housekeepers with spinal deformities (such as excessive lumbar or cervical lordosis, increased thoracic kyphosis and scoliosis), inflammatory diseases and who had a history of traumatic injury affecting musculoskeletal system were excluded from the study.

### Sample size determination and sampling procedures

The sample size was determined by using a single population proportion formula, assuming a 50% proportion of back pain, 5% margin of error and 95% confidence interval resulting in 384 hotel housekeepers. By considering possible non-response during the data collection period the final sample size increased by 10% and come up with 422 study participants. A systematic random sampling technique was used to select the study participants among the hotels. To reach the study participants we have calculated the interval size (K) by the formula K=N/n and obtained the sample interval to be 3. After selecting the first participant randomly from the first three housekeepers, every K^th^ unit (3rd housekeeper) was interviewed. The list of housekeepers was randomized before selecting every 3rd housekeeper to assure a true random sampling.

### Data collection tool and procedure

The data was collected through face to face interview data collection technique.

The low back pain among the study participants was measured by the standardized Nordic questionnaire for the analysis of musculoskeletal symptoms [23]. The questionnaire was designed to assess musculoskeletal trouble occur in a given population with consideration of in which parts of the body they are localized. The reliability of the questionnaire has been shown to be acceptable [23]. In this study, the reliability/internal consistency of the Nordic questionnaire was checked by calculating Cronbach’s alpha. Accordingly, the Cronbach’s alpha for the questionnaire was 0.77. Literature indicates that a value of Cronbach’s alpha 0.60 or greater is assumed to be acceptable [24]. Therefore, the questionnaire in this study was found to have acceptable reliability.

In addition, socio-demographic, personal and work environment characteristics of the participants were also collected.

The questionnaire was originally in the English version and it was translated to Amharic and back to English by another translator to check the consistency of the message from the question. The translation was then reviewed by professional experts. Prior to the commencement of actual data collection, the questionnaire was pretested in 42 (10%) of study subjects in Woreta town hotel housekeepers and necessary modifications were made on the tool. Six Bachelor of Science graduate of Environmental and Occupational Health and Safety’ data collectors with three supervisors were assigned for data collection.

### Operational definitions

#### Low back pain

Defined as chronic or acute aches, pain, trouble or discomfort in the lumbar or buttock areas during the 12 months preceding the completion of the questionnaire [23].

#### Body mass index (BMI)

Weight in kilograms divided by the square of the height in meters (kg/m^2^).

- Underweight = BMI < 18.50.
- Normal range = BMI b/n 18.50–24.99.
- Overweight = BMI b/n 25.00–29.99.
- Obese = BMI ≥30.00.

#### Satisfaction

The employee was considered satisfied with a job when his/her sum of generic job satisfaction scale score was 32 or above [25].

#### Physical exercise

Performing any kind of physical exercise away from the work at least two times per week and for 30 min.

### Data management and analysis

Data was checked, edited, coded and entered to Epi-info version 7.00 and exported to Statistical Package for Social Science (SPSS) version 20 for further analysis.

A chi-square test was conducted to see the association of different factors with the magnitude of low back pain. A binary logistic regression model was fitted to identify factors associated with low back pain. Low back pain was regressed against the socio-demographic, personal and work environment factors separately. Before fitting the binary logistic regression model, the goodness of the model fit test was checked by Hosmer and Lemeshow test and the assumption was satisfied (*p*-value > 0.05).

Bivariate logistic regression analysis was performed and variable with p-value < 0.20 was exported to multivariable logistic regression analysis. The significance level was obtained at 95% CI and *p*-value ≤0.05. The adjusted odds ratio was used to determine the strength of association.

### Data quality control

During the collection of data, each completed questionnaire was checked for consistency and completeness by supervisors. Throughout the course of the data collection, data collectors were supervised at each site, regular meetings were held between the data collectors and the principal investigator. Ten percent of data was double entered to check error during data entry.

## Results

All 422 completed and valid questionnaires were returned and considered for the analysis, which gives a response rate of 100%.

### Socio-demographic characteristics of the study participants

Majority 388 (91.9%) of the study participants were females. Nearly one third 131 (31%) of respondents had more than 2 years of work experience in a hotel housekeeping job. The mean (± SD) age of the respondents was 26.71 ± 4.9 years and 10 (2.4%) of participants were obese (Table 1).

**Table 1:** Socio-demographic and Personal characteristics of the study participants, Northwest Ethiopia, 2017 (*n* = 422)

Variables	Frequency (n)	Percent (%)
Sex		
Female	388	91.9
Male	34	8.1
Age (years)		
≤ 24	194	46
25–29	100	23.7
> 29	128	30.3
Marital status		
Single	206	48.8
Married	179	42.4
Divorced/Widowed	37	8.8
Employment pattern		
Permanent	378	89.6
Temporary	44	10.4
Specific Work experience in this work area		
1–2 year	291	69
> 2 year	131	31
Monthly salary		
≤ 500	96	22.7
501–1000	267	63.3
> 1000	59	14
Body mass index (BMI)		
Under weight	71	16.8
Normal weight	277	65.6
Over weight	64	15.2
Obese	10	2.4
Cigarette smoking		
None	417	98.8
Past smoker	3	0.7
Current	2	0.5
Alcohol drinking		
No	314	74.4
Yes	108	25.6

### Working condition of respondents

Above half 229 (54.3%) of respondents were made 15–30 beds per day and more than half, 221 (52.4%) of the respondents’ job involve reaching/overstretching. Moreover, 248 (58.8%) of respondents were not satisfied with their current working condition and only nearly one-fourth 99 (23.5%) of study participants received training related to health and safety (Table 2).

**Table 2:** Working conditions of the study participants, Northwest Ethiopia, 2017 (*n* = 422)

Variables	Frequency (n)	Percent (%)
Hours worked per day		
≤ 8 h	351	83.2
> 8 h	71	16.8
Number of beds making per day		
≤ 15 beds	119	28.2
15–30 beds	229	54.3
> 30 beds	74	17.5
Rest break taken per day (excluding lunch break)		
Once for < 30 min	107	25.4
Twice (30–45 min)	225	53.3
> 3 times (15 min/60 min work)	90	21.3
The job require reaching/over stretching		
No	201	47.6
Yes	221	52.4
The job require repetitive Bending		
No	204	48.3
Yes	218	51.7
Satisfaction		
No	248	58.8
Yes	174	41.2
Training related with health and safety		
No	323	76.5
Yes	99	23.5

### Prevalence of low back pain

This study revealed that the prevalence of low back pain among hotel housekeepers in Gondar town was 58.1% (95% CI: 53.6, 62.8).

**Fig. 1:**
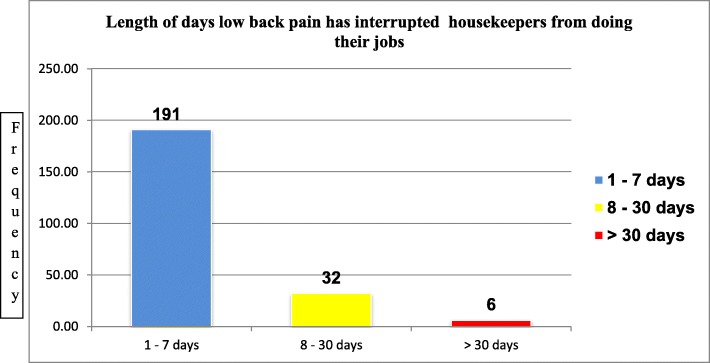
Frequency of respondents who were interrupted from doing their jobs due to low back pain, Northwest Ethiopia, 2017 (*n* = 229)

Furthermore, back pain has affected/interrupted 229 (54.3%) of respondents ability to work their normal works at least for 1 day during the last 12 months. Among these, the majority 191 (83.4%) of study participants reported low back pain has prevented them from doing their jobs one to 7 days in the past 12 months (Fig. 1).

### Factors associated with low back pain

The multivariable binary logistic regression showed that employment pattern, rest break taken, reaching/overstretching, repetitive bending, satisfaction, training related to health and safety and numbers of beds making were had a statistically significant association with low back pain (*p*-value ≤0.05). However, there was no significant association observed between sex, age, work experience, physical exercise, BMI and low back pain (Table 3).

**Table 3:** Bivariate and Multi variable logistic regression analysis of risk factors associated with low back pain among hotel housekeepers, Northwest Ethiopia, 2017 (*n* = 422)

Variables	Back pain		COR (95% CI)	AOR (95% CI)
	No	Yes		
Sex				
Male	20	14	1.00	1.00
Female	157	231	2.1(1.03, 4.29)*	1.22(0.51, 2.88)
Age (years)				
≤ 24	84	113	1.00	1.00
25–29	50	50	0.72(0.44, 1.16)	0.56(0.30, 1.02)
> 29	46	82	1.28(0.81, 2.03)	1.31(0.72, 2.36)
Specific Work experience in this work area				
1–2 year	129	162	1.00	1.00
> 2 year	48	83	1.38(0.90, 2.10)	0.99(0.59, 1.69)
Employment pattern				
Permanent	164	214	1.00	1.00
Temporary	13	31	1.83(0.93, 3.60)	3.22(1.42, 7.33)*
Physical exercise				
No	167	230	1.00	1.00
Yes	10	15	1.09(0.48, 2.48)	0.48(0.17, 1.31)
Body mass index (BMI)				
Under weight	24	47	1.00	1.00
Normal weight	122	155	0.65(0.38, 1.12)	0.76(0.39, 1.48)
Over weight	28	36	0.66(0.33, 1.32)	0.81(0.34, 1.89)
Obese	3	7	1.19(0.28, 5.02)	0.75(0.15, 3.84)
Rest break taken per day (excluding lunch break)				
Once for < 30 min	31	76	1.00	1.00
Twice (30–45 min)	87	138	0.65(0.39, 1.06)	0.79(0.44, 1.41)
> 3 times (15 min/60 min)	59	31	0.21(0.12, 0.39)**	0.49(0.24, 0.99)*
The job require reaching/over stretching				
No	130	71	1.00	1.00
Yes	47	174	6.78(4.4, 10.45)**	2.93(1.53, 5.60)**
The job require repetitive Bending				
No	125	79	1.00	1.00
Yes	52	166	5.05(3.32, 7.69)**	1.97(1.03, 3.75)*
Satisfaction				
No	69	179	1.00	1.00
Yes	108	66	0.24(0.16, 0.36)**	0.49(0.29, 0.83)*
Training related to health and safety				
No	116	207	1.00	1.00
Yes	61	38	0.35(0.22, 0.56)**	0.49(0.28, 0.87)*
Number of beds making				
≤ 15 beds	61	58	1.00	1.00
15–30 beds	98	131	1.41(0.90, 2.19)	1.25(0.73, 2.13)
> 30 beds	18	56	3.27(1.72, 6.21)**	3.19(1.50, 6.77)*

1:00 = reference, * = variable *P*-value ≤0.05, ** = *p*-value ≤0.001

This study showed that temporary employees had a higher risk of developing back pain. Hotel housekeepers with temporary employment status had 3.22 times higher odds of having low back pain when compared to permanent hotel housekeepers (AOR = 3.22, 95% CI: 1.42, 7.33). Taking short and frequent rest breaks was associated with less likelihood of developing low back pain. Respondents who had taken more than 3 times (15 min per 1 h) rest break per day had 51% less likely odds of developing low back pain compared to participants who had taken once (less than 30 min) rest break per day (AOR = 0.49, 95% CI: 0.24, 0.99).

According to this study, tasks that require reaching/overstretching were a risk factor for low back pain. Hotel housekeepers whose task requires reaching/overstretching had 2.93 times higher odds of acquiring low back pain than those whose task does not require reaching/overstretching (AOR = 2.93, 95% CI: 1.53, 5.60). Moreover, respondents whose job requires repetitive bending had 97% increased odds of having low back pain than those whose task does not require repetitive bending (AOR = 1.97, 95% CI: 1.03, 3.75).

Furthermore, respondents who were satisfied with their current job had 51% less likely odds of having low back pain when compared to those who were not satisfied (AOR = 0.49, 95% CI: 0.29, 0.83). Reduction in the odds of having low back pain was observed among housekeepers who took health and safety training. Housekeepers who have received health and safety training had 51% less probability of developing low back pain than their counterparts who didn’t receive training (AOR = 0.49, 95% CI: 0.28, 0.87). As per this study, hotel housekeepers who make > 30 beds per day had 3.19 higher odds of developing low back pain than those who make ≤15 beds per day (AOR = 3.19, 95% CI: 1.50, 6.77).

## Discussion

This study was aimed at assessing the prevalence of low back pain and its associated factors. According to this study, the prevalence of low back pain among hotel housekeepers was 58.1% [95% CI: 53.6, 62.8]. This result revealed that housekeepers are at high risk for developing low back pain. This finding is consistent with other studies that reported 60% in India among hotel industry housekeeping staff [9], 60% in Malaysia among hotel room attendants [26] and 58% in Texas among hospital housekeeper [27]. These findings corroborated the magnitude of low back trouble is high among housekeepers. On the other hand, this study result was higher than the study done among cleaners in the UK which was reported 46% [13]. The possible reason might be attributed to the better health and safety service delivered in the UK than this study area.

However, this finding is slightly lower when compared to the research reported, 63.3% among domestic servants in Egypt [28]. This might be due to all the study participants in the latter study were females who are more prone to musculoskeletal disorders [29]. In addition, the health and safety concern between the two studies settings might differ. In contrast, the finding of this study is much lower than the study done among homemakers in Lebanon (77%) [15]. The possible explanation for the difference might be in the later research other musculoskeletal symptoms were included, while only low back pain was reported in our study. Organizational difference and sex distribution among study participants might also play a role in the difference.

Employment status was found to be a significant predictor of back pain; temporary workers had higher odds of developing back pain when compared to permanent employees. The possible reason might be the difference in benefit packages in which the industry provides to permanent and temporary workers. Hence, temporary workers have limited access to basic safety training and the use of personal protective devices. Moreover, these groups of workers usually forced to do more tasks for fear of losing their job or appealing to stay on their job for more time [30–32].

The workload was associated with low back pain. In this study, the numbers of a bed made per day have had a significant association with developing low back pain. This result is supported by the study conducted in Las Vegas among hotel cleaners, which states that the odds to experience low back pain were 44% higher among workers who made 19 or more beds per day than those who made 18 or fewer [33]. Moreover, the study done in Nigeria among housekeepers identified as bed-making duties put the back in its weakest position [34]. This might be due to the fact that bed-making demands load on the musculoskeletal part of the body particularly on the back and as the number of a bed made increases the exposure period for physical exertion increases. While taking short and frequent rest breaks was associated with less likelihood of developing low back pain when compared to participants who had taken once (less than 30 min) rest break per day. The possible explanation for this might be a housekeeping task involves continuous washing and mopping of floors, bathrooms, changing sheets and towels, bed making, emptying wastebasket, and pushing heavy supply carts which all needs force exertion and repetitive awkward postures that leads high utilization of energy and musculoskeletal system for long periods without breaks. Hence, frequent rest break is crucial in the recovery of stretched muscles and energy expenditure; which can be useful in preventing low back pain. This result is supported by the report of the European agency of health and safety which stated as prolonged work without the opportunity to rest is a risk factor for MSDs [35].

According to this study jobs which require reaching/overstretching and repetitive bending was a risk factor for low back pain. Hotel housekeepers whose job requires reaching/overstretching had higher odds of acquiring back pain than those whose task does not require reaching/overstretching. This finding is supported by the research done in Egypt [28], European agency of health and safety report [6] in which awkward posture such as reaching is identified as an associated risk factor for musculoskeletal trouble including low back pain among cleaners. Moreover, respondents whose job requires repetitive bending had increased odds of having low back pain by 97% than whose task does not require repetitive bending. In concordance with this study, awkward posture or bending among female homemakers were reported as risk factors for low back pain in Lebanon [15]. This might be due to the fact that when peoples work in overstretched conditions; tendons, ligaments, joints, and muscles on the back will move out of their neutral posture and force unnecessary pressure on the back. These result in more strain and tear of soft tissues and finally increases the risk of low back pain. In addition, bending the posture with high frequency makes the workers’ back muscles and other soft tissues overstretched; which is one of the greatest contributory factors for the onset of low back pain. This is supported by the study done in Malaysia hotel room attendants and UK general cleaners on which bending had been found as an associated risk factor with back-related musculoskeletal disorders [13, 36]. Moreover, as hotel housekeepers engaged in bed making it forces the body to be in awkward posture which ultimately causes muscle strain on the back and then finally results in back pain.

Furthermore, respondents who were not satisfied with their current job had reported higher odds of having low back pain when compared to those who were satisfied. The possible reason might be those workers who were dissatisfied with the working condition and environment might develop work-related stress which leads to muscle tension and this again exacerbate the development of pain on the back. On the contrary, satisfied workers could manage the job demand and control imbalance in a better way and this lessens the likelihood to develop low back pain than their counterparts. This result is supported by the study conducted in Iran in which job dissatisfaction was strongly associated with low back pain [37] and as well in Netherland as low job satisfaction was identified as a risk factor for sickness absence due to low back pain [3].

This study revealed that taking health and safety training was important in preventing low back pain. Respondents who have received health and safety training had 51% less likely odds of developing low back pain than their counterparts who didn’t receive training. This result is in line with the study done in India among housekeepers and identified as ergonomic intervention training and advice showed a beneficial effect to reduce MSDs. This might be due to the fact that those housekeepers who had taken training might develop awareness on good practice and skill on different ergonomic interventions like using appropriate tools and equipment, frequent breaks, using correct anatomical posture and utilize this intervention which is evidenced to prevent the development of musculoskeletal disorders including low back pain [38]. In addition poor or no training was reported as a risk factor for the development of MSDs in the European agency of health and safety report [6].

### Limitations

Though this study was able to provide important data on low back pain among hotel housekeepers, several limitations are noted. While we were able to investigate associations between low back pain and important variables, causation could not be established, nor were ergonomic audits of workstations and activities undertaken. Furthermore, the lack of accompanying physical examination to strengthen/verify the self-reported symptom was the limitation of this study. In addition, since this study was a questionnaire-based cross-sectional study the possibility of recall bias could not be ruled out since more serious and recent pains or troubles remembered better than a less serious and older one. But we have tried to minimize the effect by honestly explaining the objective and significances of the study to the study participants and by using a standardized questionnaire for assessing back pain. Despite these limitations, we feel the study provides a reasonably accurate assessment of low back pain and associated risk factors among hotel housekeepers.

## Conclusion

In conclusion, this study showed a high prevalence of low back pain among hotel housekeepers.

Employment pattern, Rest break taken, Reaching/overstretching, Repetitive bending, job satisfaction, training related to health and safety and numbers of beds making were among the risk factors associated with low back pain. Therefore, adjusting organizational measures, promoting and practicing frequent rest break, avoiding overstretching and repetitive bending, improving job satisfaction and delivering ongoing safety training is among the most potent essential measures required in preventing low back pain.

## Data Availability

All data generated or analyzed during this study are included in this article. The data that support the findings of this study are also available from the corresponding author upon reasonable request.

## Abbreviations

AOR: Adjusted odds ratio
BMI: Body mass index
CI: Confidence interval
COR: Crude odds ratio
LBP: Low back pain
MSDs: Musculoskeletal disorders
SPSS: Statistical package for social science
UK: United Kingdom
